# Comparison of quantification algorithms for circulating cell-free DNA methylation biomarkers in blood plasma from cancer patients

**DOI:** 10.1186/s13148-017-0425-4

**Published:** 2017-12-01

**Authors:** Luka de Vos, Heidrun Gevensleben, Andreas Schröck, Alina Franzen, Glen Kristiansen, Friedrich Bootz, Dimo Dietrich

**Affiliations:** 1 Department of Otolaryngology, University Hospital Bonn, Head and Neck Surgery, Sigmund-Freud-Str. 25, 53127 Bonn, Germany; 2 Institute of Pathology, University Hospital Bonn (UKB), Bonn, Germany

**Keywords:** Free-circulating cell-free DNA, DNA methylation, *SHOX2*, *SEPT9*, Diagnostic accuracy, Risk stratification, Quantitative real-time PCR, ΔΔCT-method, ΔCT-method, Quasi-digital PCR

## Abstract

**Background:**

*SHOX2* and *SEPT9* methylation in circulating cell-free DNA (ccfDNA) in blood are established powerful and clinically valuable biomarkers for diagnosis, staging, prognosis, and monitoring of cancer patients. The aim of the present study was to evaluate different quantification algorithms (relative quantification, absolute quantification, quasi-digital PCR) with regard to their clinical performance.

**Methods:**

Methylation analyses were performed in a training cohort (141 patients with head and neck squamous cell carcinoma [HNSCC], 170 control cases) and a testing cohort (137 HNSCC cases, 102 controls). DNA was extracted from plasma samples, bisulfite-converted, and analyzed via quantitative real-time PCR. *SHOX2* and *SEPT9* methylations were assessed separately and as panel [mean_*SEPT9*/*SHOX2*_] using the ΔCT method for absolute quantification and the ΔΔCT-method for relative quantification. Quasi-digital PCR was defined as the number of amplification-positive PCR replicates. The diagnostic (sensitivity, specificity, area under the curve (AUC) of the receiver operating characteristic (ROC)) and prognostic accuracy (hazard ratio (HR) from Cox regression) were evaluated.

**Results:**

Sporadic methylation in control samples necessitated the introduction of cutoffs resulting in 61–63% sensitivity/90–92% specificity (*SEPT9*/training), 53–57% sensitivity/87–90% specificity (*SHOX2*/training), and 64–65% sensitivity/90–91% specificity (mean_*SEPT9*/*SHOX2*_/training). Results were confirmed in a testing cohort with 54–56% sensitivity/88–90% specificity (*SEPT9*/testing), 43–48% sensitivity/93–95% specificity (*SHOX2*/testing), and 49–58% sensitivity/88–94% specificity (mean_*SEPT9*/*SHOX2*_/testing). All algorithms showed comparable cutoff-independent diagnostic accuracy with largely overlapping 95% confidence intervals (*SEPT9*: AUC_training_ = 0.79–0.80; AUC_testing_ = 0.74–0.75; *SHOX2*: AUC_training_ = 0.78–0.81, AUC_testing_ = 0.77–0.79; mean_*SEPT9*/*SHOX2*_: AUC_training_ = 0.81–0.84, AUC_testing_ = 0.80). The accurate prediction of overall survival was possible with all three algorithms (training cohort: HR_*SEPT9*_ = 1.23-1.90, HR_*SHOX2*_ = 1.14-1.85, HR_mean*SEPT9*/*SHOX2*_ =1.19-1.89 ; testing cohort: HR_*SEPT9*_ =1.22-1.67, HR_*SHOX2*_ = 1.15-1.71, HR_mean*SEPT9*/*SHOX2*_ = 1.12-1.77).

**Conclusion:**

The concordant clinical performance based on different quantification algorithms allows for the application of various diagnostic platforms for the analysis of ccfDNA methylation biomarkers.

**Electronic supplementary material:**

The online version of this article (10.1186/s13148-017-0425-4) contains supplementary material, which is available to authorized users.

## Background

Cancer is the second most common cause of death in the USA, therefore posing a major health burden. Approximately 1,688,780 newly diagnosed cases are expected in 2017 [1]. Consequently, there is a pressing need to identify biomarkers that might help to address key clinical questions and improve patient outcome. Biomarkers derived from liquid biopsies appear to be particularly suitable, since they can be obtained minimally invasive. Circulating cell-free DNA (ccfDNA), which can be detected in the bloodstream of patients with advanced but also early stages of various malignancies [2], constitutes a promising liquid biopsy cancer biomarker [3] and is suitable for diagnosis, prognosis, and the identification of occult tumor recurrence [4–6]. Cancer-specific changes, e.g., mutations or aberrant methylation patterns, facilitate the robust discrimination between non-tumorous and tumorous ccfDNA [7].

Aberrant DNA methylation, a hallmark of cancer, frequently occurs in defined gene regions in most tumor entities and is chemically stable [7–10]. The methylation biomarkers with the highest level of validation in bodily fluid are *SHOX2* and *SEPT9*. In lung cancer, the reliability of *SHOX2* as a biomarker has been confirmed in bronchial aspirates and plasma [11–14]. Hypermethylated *SEPT9*, in addition, has been validated as a highly sensitive and specific biomarker for the detection of colorectal cancer in plasma and has recently received approval as a screening marker by the Food and Drug Administration (FDA) [15–19]. Furthermore, both *SEPT9* and *SHOX2* are diagnostic and prognostic tools for the distinction between malignant and paramalignant ascites and pleura effusions [20, 21]. Recently, a large prospective cohort study showed that both *SEPT9* and *SHOX2* are clinically valuable for diagnosis, staging, prognosis, and monitoring for patients suffering from head and neck squamous cell carcinoma (HNSCC) [3]. Since not all tumors presented with elevated methylation levels in both genes, the paneling of *SEPT9* and *SHOX2* turned out to be advantageous compared to the separate analysis of each marker [3]. Especially with regard to the diagnostic application, the combined analyses allowed for a reduction of false-positive results [3].

The application of methylated ccfDNA as a cancer biomarker, however, poses significant challenges. Firstly, ccfDNA concentrations in body fluids are considerably low, and the methylated portion is usually in the range of single genome copies. Hence, relatively high bodily fluid volumes are required, and DNA analysis needs to be highly sensitive and specific [22–24]. Methylated ccfDNA can be obtained either from plasma or from serum. The latter shows higher concentrations of ccfDNA, presumably due to the clotting process by which most leucocytes are lysed and release DNA [23, 25]. Secondly, an unspecific and low sporadic methylation in healthy individuals can occur, necessitating a cutoff above which samples are defined as methylation positive [21, 26]. While some healthy individuals might have elevated biomarker levels (false-positives), some cancer patients might not (false-negatives). Not only the underlying biological process but also the evaluation algorithm has an influence on the clinical performance of methylation biomarkers. In previous analyses of *SEPT9*, for instance, the sensitivity and specificity were heavily influenced by the evaluation algorithm [14, 27]. Accordingly, the algorithm has been discussed in the past in order to increase the performance of the test.

Quantitative real-time PCR is well established for DNA methylation analyses [3, 11–14, 20, 21, 24]. Quantification can be carried out either relatively referring to the total amount of DNA in the sample or absolutely by quantifying the number of genome equivalents in a given sample volume [28]. In order to detect the exact amount of methylated ccfDNA from the sample, a known quantity of reference DNA, which is compared to the unknown quantity from the sample, is required (reference sample). The absolute value can be calculated by the ΔCT-method. Estimating that a diploid genome weights about 6.6 pg, the absolute values can be converted into genome equivalents. For relative quantification, the methylated portion of ccfDNA is referred to the total DNA amount in the sample and can be calculated by the ΔΔCT-method [29]. Furthermore, a qualitative method based of the counts of positive PCRs can be applied (digital PCR). In accordance with a prior study, in which rare DNA variants were detected, this digital counting of PCR replicates with methylated ccfDNA can be called “quasi-digital PCR” [30]. On the basis of the distribution of positive and negative reactions, Poisson statistics can predict the amount of methylated ccfDNA in the sample [31]. Most studies on *SEPT9* in colorectal cancer employed a qualitative method, especially the triplicate PCR method [15, 17–19, 27]. Here, the sample is divided into three real-time PCR reactions, with some aliquots containing methylated gene copies while others do not. The number of positive reactions is then counted and represents the total amount of methylation in the sample. In different prior studies, the sample was considered positive, if either one out of three (1/3 algorithm) or two out of three (2/3 algorithm) replicates showed a reaction [27, 32–34].

Currently, no explicit recommendation exists as to whether quantitative or qualitative analyses are supposed to be applied when using methylated ccfDNA as a biomarker for cancer. Depending on the study, different evaluation methods, mostly relative quantification and quasi-digital PCR, were used [3, 11–15, 17–21, 27, 29, 31]. The aim of the present study was to compare the three different algorithms in order to improve the clinical performance of methylated ccfDNA as a biomarker with the example of *SEPT9* and *SHOX2*. This study was carried out based on a reevaluation of recently published data [3] from a prospective cohort study including HNSCC patients. According to the original study, analysis was performed in a training/testing design in order to avoid overfitting. Methylation of *SEPT9* and *SHOX2* in plasma was analyzed with three different algorithms (absolute quantification, relative quantification, and quasi-digital PCR), and the diagnostic accuracy of all three algorithms was compared. Furthermore, the patients’ clinical follow-up was updated allowing for an accurate prognostic analysis.

## Methods

### Patient cohort and clinical endpoints

A training cohort of 137 HNSCC patients and 170 control patients with benign diseases was included in the present study. Of these, 137 HNSCC patients and 122 control patients had been enrolled in the course of a previous study [3]. Forty-eight additional control patients were included in the training cohort, and the clinical follow-up was updated. For validation of the results, the same testing cohort as in the previous study was included (141 HNSCC patients, 102 control patients), and overall survival data were updated. For detailed patients’ characteristics see Additional file 1. All patients were treated at the University Hospital Bonn between 2012 and 2017. Control patients had no history of SCC and were free of any other malignancy in at least 3 years [3]. A plasma sample was taken prior to any treatment [3].

The study protocol was approved by the ethics committee of the University Hospital Bonn (vote no. 224/12). All patients provided written informed consent. The clinical study endpoint was death by any cause, and time-to-event (time-to-death) was defined as overall survival.

### Plasma preparation and *SHOX2* and *SEPT9* methylation quantification

Blood samples were taken in EDTA-stabilized collection tubes. A total of 3 ml of plasma was prepared within 2 h as previously described [3]. Bisulfite DNA was split into six parts, and each part was applied to the *SHOX2*/*SEPT9*/*ACTB* triplex quantitative methylation-specific real-time PCR. A CpG-free region within the gene actin beta (*ACTB*) was chosen to quantify methylation independently ccfDNA, representing the total DNA [20]. A calibrator sample (bisulfite converted artificially methylated human DNA) was analyzed in triplicate (3 ng each) within each PCR run. Quantitative methylation levels for *SEPT9* and *SHOX2* were calculated with the ΔCT and ΔΔCT methods.

Relative quantification was carried out using the ΔΔCT method as follows: ΔΔCt_Sample_ = ΔCt_Sample_ − ΔCt_Calibrator_ with ΔCt_Sample_ = Ct_Sample(*ACTB*)_ − Ct_Sample(*SEPT9* or *SHOX2*)_ and ΔCt_Calibrator_ = Ct_Calibrator(*ACTB*)_ − Ct_Calibrator(*SEPT9* or *SHOX2*)_. Percent methylation levels were calculated (Methylation_Sample_ = 100% • 2^ΔΔCTSample^ [29]) for each of the six PCR replicates and the mean was computed.

Absolute quantification was carried out with the ΔCT-method: ΔCt_Sample_ = Ct_Sample(*ACTB*)_ − Ct_Sample(*SHOX2* or *SEPT9*)_. Based on the quantity of the calibrator, absolute values were calculated: Methylated DNA [ng] = 3 ng × 2^ΔCT^. The number of haploid genome equivalents was calculated: $\mathrm{Genome} \mathrm{equivalent}\mathrm{s}=\mathrm{DNA}\ \left[ ng\right]/\frac{3.3\times {10}^{-3}\ \mathrm{DNA}\ \left[ ng\right]\ }{\mathrm{Genomeequivalent}}$.

For qualitative analysis (quasi-digital PCR), the number of replicates showing a reaction was counted.

### Statistical analyses

The area under the curve (AUC) of the receiver operating characteristic (ROC) was computed in order to describe the diagnostic accuracy. Sensitivity and specificity were calculated. Kaplan-Meier analyses and univariate Cox proportional hazard analysis were conducted to investigate the overall survival. In order to improve comparability between quantitative and qualitative analyses, a logarithmic scale was performed for relative methylation, absolute methylation, and positive counts. Methylation levels below 0.01% in quantitative analyses were set to 0.01% for a logarithmic illustration. In quasi-digital PCR, values below one positive replicate were set to one by adding one to all six values. Logarithmic data were used to perform Cox analysis. *P* values < 0.05 were considered statistically significant. *P* values refer to the log-rank (Kaplan-Meier) and Wald tests (Cox proportional hazards analysis), respectively. Ninety-five percent confidence intervals were reported. For the combination of *SEPT9* and *SHOX2* (mean_*SEPT9/SHOX2*_), methylation levels were mean averaged: mean_*SEPT9/SHOX2*_ = [(*SEPT9 + SHOX2*)/2] [3].

## Results

### Evaluation of the diagnostic accuracy: Training cohort

**Fig. 1:**
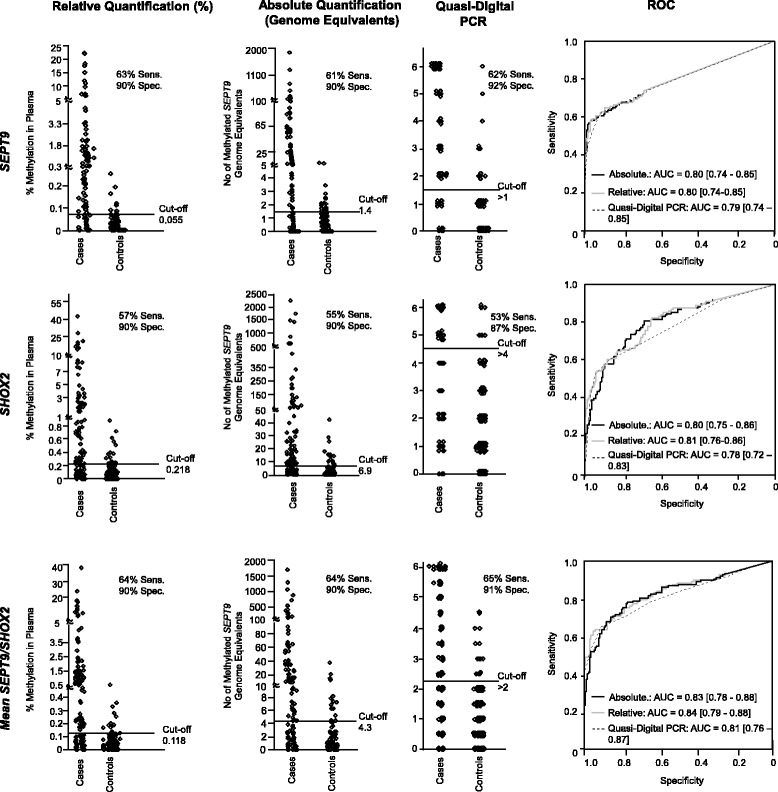
Training cohort. Methylation levels of *SEPT9*, *SHOX2*, and mean_*SEPT9*/*SHOX2*_ in plasma of HNSCC (*n* = 137) and control (*n* = 170) patients in relative quantification, genome equivalents (absolute quantification), and quasi-digital PCR with sensitivity (sens.), specificity (spec.), and cutoffs. Receiver Operating Characteristics with AUCs of HNSCC and control patients

As sporadic methylation also occurs in blood plasma from patients without a malignant disease, cutoffs are required in order to classify samples as test-positive and test-negative. In the present study, cutoffs were defined that resulted in test-negativity in 90% of control patients (specificity). According to Schröck et al. [3], *SEPT9* and *SHOX2* were analyzed separately and in combination (averaged, mean_*SEPT9/SHOX2*_). Methylation levels below 0.055% (*SEPT9*), 0.218% (*SHOX2*), and 0.118% (mean_*SEPT9/SHOX2*_) were considered test-negative (Fig. 1). As seen in Fig. 1, more than 1.4 (4.68 pg) gene copies of *SEPT9*, 6.9 (22.67 pg) of *SHOX2*, and on average 4.3 (14.27 pg) copies of both markers per sample were considered test-positive. Since the PCR analysis was performed in six single reactions per sample, the quasi-digital PCR only took on seven distinct states (0–6 PCR replicates positive). Accordingly, a determination of specificity of exactly 90% was not achievable. Specificities of 92% for *SEPT9*, 87% for *SHOX2*, and 91% for mean_*SEPT9/SHOX2*_ were accomplished by > 1 (*SEPT9*), > 4 (*SHOX2*), and > 2 (mean_*SEPT9/SHOX2*_) out of 6 positive PCR replicates.

*SEPT9* showed a low basal methylation level in blood from individuals without a malignant disease. This was indicated by the low number of positive PCR reactions when analyzing blood from the control patients (*n* = 115 with zero; *n* = 41 with one; and only *n* = 14 with more than one positive PCR). Due to the low background methylation, similar performances of all three algorithms were to be expected. Accordingly, sensitivity (63% for relative, 61% for absolute quantification, 62% for quasi-digital PCR), specificity (90, 90, and 92%, respectively), and AUCs (AUC = 0.80/0.80/0.79) were comparable with identical 95% confidence intervals (all: 95%CI [0.74–0.85]; Fig. 1). Quasi-digital PCR showed the same AUC, but in fact, the specificity was slightly higher (92%).

*SHOX2*, in contrast, had higher levels of background methylation indicated by the high number of positive PCR reactions (*n* = 48 with zero; *n* = 45 with one; and *n* = 77 with more than one positive PCR) in control patients. Therefore, a higher cutoff was required (more than four out of six PCR reactions positive). Because of the increased number of positive PCR reactions in control patients, a lower sensitivity (53%) and specificity (87%) were seen in the qualitative analysis (AUC = 0.78, 95% CI [0.72–0.83]). However, all algorithms showed similar AUCs with highly overlapping 95% confidence intervals. Relative quantification showed a slightly higher sensitivity (sensitivity = 57%) compared to absolute quantification (sensitivity = 55%) at the same defined specificity (90%).

As shown by Schröck and co-workers [3], mean_*SEPT9/SHOX2*_ gave best results in all three algorithms compared to the particular algorithm for either biomarker separately. As a result of the higher sensitivity for *SHOX2* in relative quantification, best performance of relative quantification for the mean_*SEPT9*/*SHOX2*_ was to be expected. In fact, absolute and relative quantification showed equal results (both: sensitivity = 64%, specificity = 90%, AUC_relative_ = 0.84; 95% CI_relative_ [0.79–0.88], AUC_absolute_ = 0.83, 95% CI_absolute_ [0.78–0.88]). Both biomarkers evaluated by quasi-digital PCR showed highest performance with a sensitivity of 65% at 91% specificity (AUC = 0.81, 95%CI [0.76–0.87]).

### Validation of the diagnostic accuracy: testing cohort

**Fig. 2:**
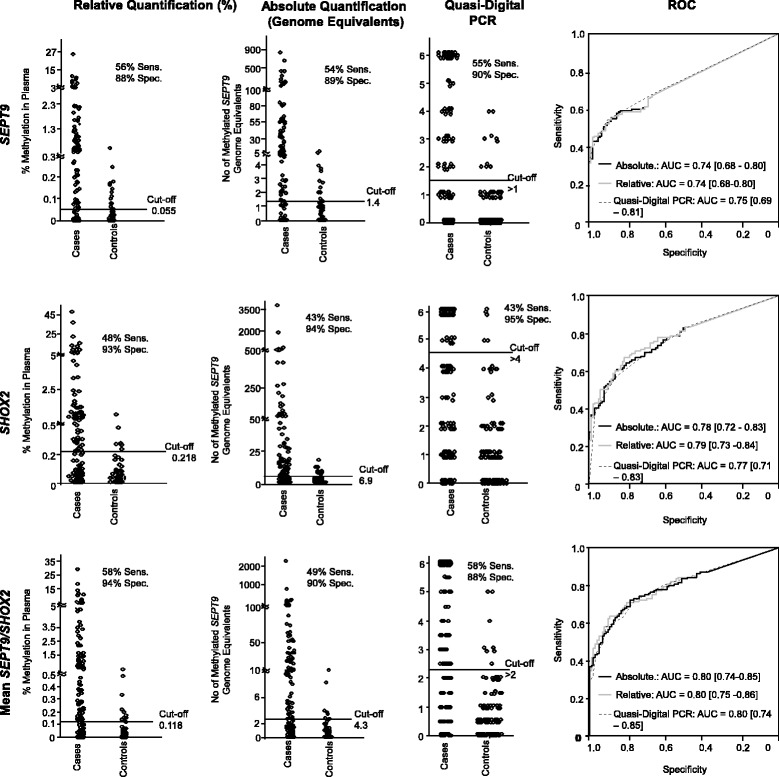
Testing cohort. Methylation levels of *SEPT9*, *SHOX2*, and mean_*SEPT/SHOX2*_ in plasma of HNSCC (*n* = 141) and control (*n* = 102) patients in relative quantification, genome equivalents (absolute quantification), and quasi-digital PCR with sensitivity (sens.), specificity (spec.), and cutoffs. ROC curves with AUCs of HNSCC and control patients

The high performance of the biomarkers was validated in the testing cohort (Fig. 2). In concordance with the results from the training cohort, all algorithms gave similar results with similar AUCs and overlapping confidence intervals. Relative quantification resulted in a slightly higher sensitivity (56%) for *SEPT9* compared to quasi-digital PCR (55%) at lower specificity (88% for relative quantification compared to 90% for quasi-digital PCR). Relative quantification also appeared to be the algorithm with highest accuracy for *SHOX2* (sensitivity = 48%, specificity = 93%, AUC = 0.79, 95% CI [0.73–0.84]). Compared to both biomarkers separately, the mean_*SEPT9*/*SHOX2*_ showed highest sensitivity and specificity in relative quantification (sensitivity = 58%, specificity = 94%, AUC = 0.80, 95% CI [0.75–0.86]).

### Prognostic value: training cohort

In concordance with the previous study, 129 HNSCC patients were included in the survival analysis [3]. Both biomarkers were used as continuous values that were converted to a logarithmic scale prior to analysis in order to improve the comparability between the quantitative scales (relative and absolute quantification) and the qualitative scale (quasi-digital PCR). For example, methylation values in plasma varied from 0% to more than 20%, whereas quasi-digital PCR, in contrast, only varied in a range of 0–6 positive replicates. All data were therefore transformed into a logarithmic scale, and Cox proportional hazards analysis was carried out with the converted data.

**Fig. 3:**
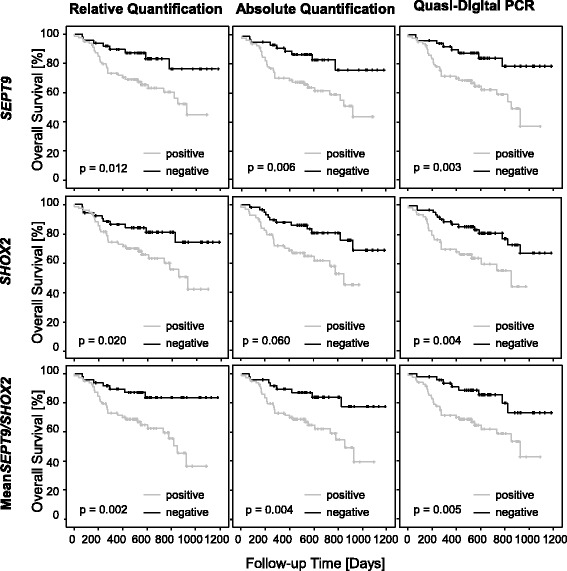
Training cohort. Kaplan-Meier analysis of overall survival in HNSCC patients (*n* = 129). Patients are stratified according to *SHOX2* and *SEPT9* plasma methylation levels. Plasma methylation levels were quantified using relative, absolute quantification, and quasi-digital PCR and dichotomized based on cutoffs that resulted in specificities and sensitivities as seen in Figs. 1 and 2. Cutoff values for positive (above cutoff) and negative (below cutoff) classification: *SEPT9*, *SHOX2*, and mean_*SEPT9/SHOX2*_. Cutoffs for relative quantification were *SEPT9* = 0.055%, *SHOX2* = 0.281%, and mean_*SEPT9/SHOX2*_ = 0.118%; for absolute quantification *SEPT9* = 4.68 pg, *SHOX2* = 22.7 pg, and mean_*SEPT9/SHOX2*_ = 14.3 pg; for quasi-digital PCR for *SEPT9* > 2, for *SHOX2* > 4 and for mean_*SEPT9/SHOX2*_ > 2 positive PCR reactions

All algorithms for the evaluation of *SEPT9*, *SHOX2*, and mean_*SEPT9/SHOX2*_ turned out to be valuable for the prognosis of patients suffering from HNSCC and all with a similarly high power. Patients with elevated biomarker levels had a significantly increased hazard of death (Fig. 3, Table 1). All algorithms showed similar results with overlapping confidence intervals. Hazard ratios for *SEPT9*, *SHOX2*, and mean_*SEPT9/SHOX2*_ using relative quantification (*SEPT9*: HR_relative_ = 1.23, 95% CI [1.11–1.35]; *SHOX2*: HR_relative_ = 1.14, 95% CI [1.03–1.26]; mean_*SEPT9/SHOX2*_: HR_relative_ = 1.19, 95% CI [1.07–1.32]) were slightly smaller compared to absolute quantification (*SEPT9*: HR_absolute_ = 1.27, 95% CI [1.14–1.42]; *SHOX2*: HR_absolute_ = 1.16, 95% CI [1.03–1.31]; mean_*SEPT9/SHOX2*_: HR_absolute_ = 1.22, 95% CI [1.08–1.37]). Highest hazard ratios for all biomarkers were found with quasi-digital PCR (*SEPT9*: HR_quasi-digital_ = 1.90, 95% CI [1.34–2.69]; *SHOX2*: HR_quasi-digital_ = 1.85, 95% CI [1.18–2.91]; mean_*SEPT9/SHOX2*_: HR_quasi-digital_ = 1.89, 95% CI [1.28–2.79]). However, this was not followed by the corresponding *p* values. All *p* values showed similar results for *SEPT9* (all: *p* < 0.001), *SHOX2* (*p*_relative_ = 0.009; *p*_absolute_ = 0.013, *p*_quasi-digital_ = 0.008), and mean_*SEPT9/SHOX2*_ (all: *p* = 0.001) indicating a similar prognostic performance for all algorithms.

**Table 1:** Diagnostic and prognostic performance of *SHOX2* and *SEPT9* hypermethylation

Cohort	Biomarker	Quantification method	Sens. (%)	Spec. (%)	AUC	Hazard ratio	*P* value	95% CI
Training								
	*SEPT9*	Relative	63	90	0.80	1.23	< 0.001	1.11–1.35
		Absolute	61	90	0.80	1.27	< 0.001	1.14–1.42
		Quasi-digital PCR	62	92	0.79	1.90	< 0.001	1.34–2.69
	*SHOX2*	Relative	57	90	0.81	1.14	0.009	1.03–1.26
		Absolute	55	90	0.80	1.16	0.013	1.03–1.31
		Quasi-digital PCR	53	87	0.78	1.85	0.008	1.18–2.91
	Mean_*SEPT9/SHOX2*_	Relative	64	90	0.84	1.19	0.001	1.07–1.32
		Absolute	64	90	0.83	1.22	0.001	1.08–1.37
		Quasi-digital PCR	65	91	0.81	1.89	0.001	1.28–2.79
Testing								
	*SEPT9*	Relative	56	88	0.74	1.22	0.002	1.07–1.38
		Absolute	54	89	0.74	1.35	0.001	1.45–1.59
		Quasi-digital PCR	55	90	0.75	1.67	0.010	1.13–2.45
	*SHOX2*	Relative	48	93	0.79	1.15	0.045	1.00–1.31
		Absolute	43	94	0.78	1.24	0.007	1.06–1.44
		Quasi-digital PCR	43	95	0.77	1.71	0.032	1.05–2.80
	Mean_*SEPT9/SHOX2*_	Relative	58	94	0.80	1.12	0.006	1.06–1.39
		Absolute	49	90	0.80	1.30	0.001	1.11–1.53
		Quasi-digital PCR	58	88	0.80	1.77	0.009	1.15–2.72

Clinical performance was evaluated in HNSCC patients included in a training and a testing cohort. Methylation levels were calculated using three different algorithms (relative, absolute quantification, and quasi-digital PCR). Sensitivities (sens.), specificities (spec.), Area Under the Curve (AUC) of the Receiver Operating Characteristic, and Cox Proportional Hazards with univariate analysis of *SEPT9*, *SHOX2*, and mean_*SEPT9*/*SHOX2*_, corresponding *p* values, and 95% confidence intervals are reported

### Validation of the prognostic value: testing cohort

**Fig. 4:**
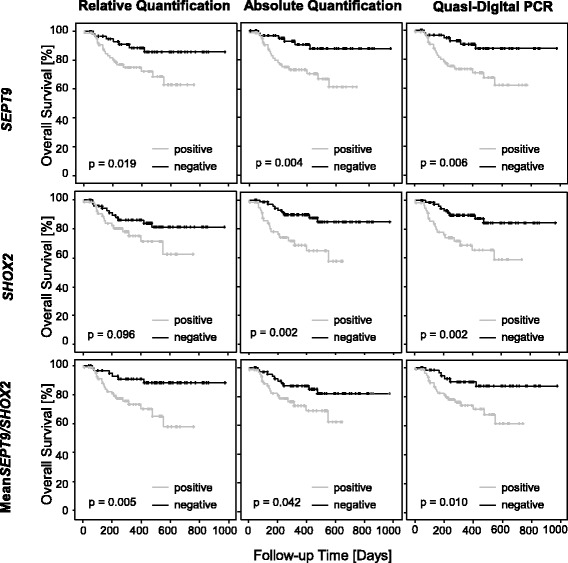
Testing cohort. Kaplan-Meier analysis of overall survival in HNSCC patients (*n* = 137). Patients are stratified according to *SHOX2* and *SEPT9* plasma methylation levels. Plasma methylation levels were quantified using relative, absolute quantification and quasi-digital PCR and dichotomized based on cutoffs established in the training cohort. Cutoff values for positive (above cutoff) and negative (below cutoff) classification: *SEPT9*, *SHOX2*, and mean_*SEPT9/SHOX2*_. Cutoffs for relative quantification were *SEPT9* = 0.055%, *SHOX2* = 0.281%, and mean_*SEPT9/SHOX2*_ = 0.118%; for absolute quantification *SEPT9* = 4.68 pg, *SHOX2* = 22.7 pg, and mean_*SEPT9/SHOX2*_ = 14.3 pg; for quasi-digital PCR for *SEPT9* > 2, for *SHOX2* > 4, and for mean_*SEPT9/SHOX2*_ > 2 positive PCR reactions

In accordance with the training cohort, all markers were significantly prognostic in all algorithms and all gave similar results (Fig. 4, Table 1). In Cox proportional hazards analysis, quasi-digital PCR showed highest hazard ratios for *SEPT9*, *SHOX2*, and mean_*SEPT9/SHOX2*_ with only small differences in confidence intervals (*SEPT9*: HR_quasi-digital_ = 1.67, 95% CI [1.13–2.45]; *SHOX2*: HR_quasi-digital_ = 1.71, 95% CI [1.05–2.80]; mean_*SEPT9/SHOX2*_: HR_quasi-digital_ = 1.77, 95% CI [1.15–2.72]). *P* values again did not concur. The smallest *p* value was found for *SEPT9* (*p* = 0.001), *SHOX2* (*p* = 0.007), and mean_*SEPT9/SHOX2*_ (*p* = 0.001) in absolute quantification.

## Discussion

Hypermethylation of the *SHOX2* and *SEPT9* gene loci in ccfDNA has been known as valuable diagnostic and prognostic tests for patients suffering from HNSCC and other cancers [3, 7, 11–21, 27, 29, 32–34]. The performance of methylation biomarkers, however, is strongly dependent on the algorithm employed [15, 18, 19, 27, 33, 34]. The aim of the present study was to compare qualitative (quasi-digital PCR) and quantitative (absolute and relative quantification) evaluation methods in order to determine the most suitable algorithm. Analysis of both *SEPT9* and *SHOX2* in different algorithms led to the finding that differences in sensitivity and specificity examined with relative quantification, absolute quantification, and quasi-digital PCR were only marginal.

*SEPT9* and *SHOX2* both have been shown to be strong and valid biomarkers in several cancer entities. Many studies have been carried out in order to examine their potential in the clinical management of cancer patients [3, 14–21]. In all studies, methylation analysis was based on PCR. While some studies used quantitative methods like the 1/3 or the 2/3 algorithms [19, 27, 32, 33], others quantified both biomarkers relatively in reference to the amount of total DNA [3, 20, 21]. This led to the following questions: (a) to what extend can the clinical performance of biomarkers be compared between studies using quasi-digital PCR and studies using relative quantification and (b) which of the algorithms is more suitable. It has been shown previously that the clinical performance of methylation biomarkers is heavily influenced by the algorithm employed for analysis. In a prospective cohort study of *SEPT9* in colorectal cancer screening, for instance, sensitivity could be increased from 48.2 to 63.9% while specificity simultaneously decreased from 91.5 to 88.4% by using three instead of only two PCR replicates [15]. Not only the number of PCR replicates but also the number of positive PCR reactions to call the sample positive had an influence in prior studies. In reports comparing the 1/3 algorithm to the 2/3 algorithm, the 1/3 algorithm resulted in a higher sensitivity (75%) compared to the 2/3 algorithm (57%) but also lead to more false-positive results (specificity: 87% for the 1/3 algorithm vs. 98% for the 2/3 algorithm) [19]. This finding was further confirmed in other studies [27, 31–33]. In the past, such comparisons have been made with algorithms all based on quasi-digital PCR but not between quasi-digital PCR and quantitative methods. The present study revealed that both *SHOX2* and *SEPT9* show an equally strong performance using absolute quantification, relative quantification, and quasi-digital PCR and can therefore be considered extraordinary robust biomarkers. This aspect allows for the application of different analytical platforms that all exhibit advantages and disadvantages. One major advantage of quasi-digital PCR is its simplicity and feasibility. The digital algorithm with three PCR replicates was implemented in most studies of *SEPT9*. In the past, it was found that an increase in PCR replicates substantially enhanced the sensitivity at the expense of a slight reduction of specificity [15]. This led to the consideration that clinical performance (accuracy) of methylation biomarkers could further be increased by the application of additional PCR replicates. Thus, six replicates were used in the present study, assuming that the representation of the overall methylated ccfDNA in the sample may be more accurate.

Even though samples are retrieved under highly standardized conditions, ccfDNA can increase in vitro. In an earlier study evaluating patients with bone marrow transplants, most of the ccfDNA in the bloodstream was shown to originate from the hematopoietic system. The patients under investigation had received bone marrow transplants from individuals of the other gender, and ccfDNA was related by analyzing the y-chromosome. Additionally, serum and plasma samples were compared, and serum contained 14-fold higher concentrations of ccfDNA compared to plasma, indicating that the additional ccfDNA was derived from lysed leucocytes. Besides, long storage times also increase concentrations of ccfDNA in plasma [35]. Samples with long times of storage before plasma preparation could therefore benefit from absolute quantification rather than from relative quantification, since it is independent of in vitro increased total DNA values. Especially if sample quality cannot be reproduced, evaluation of methylation could benefit from absolute quantification. In contrast to relative quantification in reference to the total DNA present in the sample, absolute quantification is not influenced by increased total DNA originating from leucocytes. Because methylated ccfDNA might predominantly originate from the tumor rather than from leucocytes, absolute quantification could be an ideal algorithm for the analysis of methylation biomarkers from serum. As the present study shows that absolute quantification, quasi-digital PCR, and relative quantification perform equally, one perspective for the future could be the application of absolute quantification to serum analysis of methylated ccfDNA. However, because the short half-life of 10–15 min of ccfDNA in blood allows for no biomarker accumulation in vivo [36, 37], concentrations, especially of tumorous ccfDNA, are always low. This dynamic might limit the performance of absolute quantification in contrast to relative quantification. In the future, an internal control might help to overcome such issues, for example as recently described [38]. This internal control, when chosen with a chemical quality similar to the analyzed ccfDNA, could improve validating the analytical sensitivity, which is especially important for analytes with low target concentrations like methylation [38]. The internal control could also serve as a reference standard for relative quantification, being independent of increased ccfDNA from leucocytes occurring during plasma preparation.

In the present study, *SEPT9*, *SHOX2*, and mean_*SEPT9/SHOX2*_ proved to be significant prognostic factors for overall survival in HNSCC patients. Elevated biomarker levels were associated with a worse overall survival in both the training and the testing cohort. Transferring methylation levels and the count of positive PCR replicates onto a logarithmic scale made them comparable. For all biomarkers, quasi-digital PCR showed highest hazard ratios. However, even in a logarithmic scale, relative quantification showed values between 0% and about 22% methylation (training cohort) and 25% methylation (testing cohort), whereas in quasi-digital, PCR results only ranged from zero to six positive replicates. This limited the comparability of the methods applied. Because all algorithms were tested in plasma of the same patients, comparing *p* values had a higher explanatory power compared to the hazard ratios. The comparison of the *p* values did not show a clear direction towards which algorithm performed best for prognostic purposes. In conclusion, prognosis of patients with HNSCC, *SEPT9*, *SHOX2*, and mean_*SEPT9/SHOX2*_ can be analyzed either with relative or absolute quantification or with quasi-digital PCR.

In the present study, only HNSCC patients who have been enrolled and analyzed within a previous study were included [3]. Hence, it needs to be emphasized that the current study is not suited and does not aim to validate the clinical performance of the biomarkers that have been described by Schröck and colleagues earlier [3]. This study rather showed the robustness of the methylation biomarkers. In the future, methylation analysis of *SEPT9* and *SHOX2* can be applied to various different platforms, for example real-time PCR platform, digital PCR, or next generation sequencing. It could even be applied to end-point PCR or a point of care platform.

## Conclusions

In summary, quantitative and qualitative evaluation methods are equally suitable for both *SEPT9* and *SHOX2* confirming the robustness and reliability of both biomarkers. The concordance of the evaluation algorithms further allows for the application of different platforms when working with methylation biomarkers.

## Additional file

Additional file 1: Baseline characteristics and clinicopathological variables. Table with characteristics of HNSCC and control patients examined in the training and the testing cohort (training cohort: *n* = 137 HNSCC patients, *n* = 170 controls; testing cohort: *n* = 141 HNSCC patients, *n* = 102 controls). For all patients, age, gender, and smoking and drinking habits are reported. For HNSCC patients, additional tumor characteristics referring to the TNM staging system are listed. (DOCX 30 kb)

## Abbreviations

*ACTB*: Actin beta
AUC: Area under the curve
ccfDNA: Circulating cell-free DNA
CI: Confidence interval
CT: Threshold cycle
HNSCC: Head and neck squamous cell carcinoma
HR: Hazard ratio
PCR: Polymerase chain reaction
ROC: Receiver operating characteristic
*SEPT9*: Septin 9
*SHOX2*: Short stature homeobox 2
