# Supplementary material for: Comparison of quantification algorithms for circulating cell-free DNA methylation biomarkers in blood plasma from cancer patients
Source: Clin Epigenetics. 2017 Dec 1;9:125. doi: 10.1186/s13148-017-0425-4 (PMC5709918; doi:10.1186/s13148-017-0425-4)
Supplement: Additional file 1: — Baseline characteristics and clinicopathological variables. Table with characteristics of HNSCC and control patients examined in the training and the testing cohort (training cohort: n = 137 HNSCC patients, n = 170 controls; testing cohort: n = 141 HNSCC patients, n = 102 controls). For all patients, age, gender, and smoking and drinking habits are reported. For HNSCC patients, additional tumor characteristics referring to the TNM staging system are listed. (DOCX 30 kb) [file 13148_2017_425_MOESM1_ESM.docx]

**Additional Table 1: Baseline characteristics and clinicopathological variables.** Characteristics and clinicopathological variables of HNSCC and control patients included into the training (n=137 HNSCC and n=170 control patients) and testing cohorts (n=141 HNSCC and n=102 control patients).

|  | **Training Cohort** | | | **Testing Cohort** | | |
| --- | --- | --- | --- | --- | --- | --- |
|  | **HNSCC patients*** |  | **Controls**** |  | **HNSCC Patients*** | **Controls*** |
| **Total** | 137 (100%) |  | 170 (100%) |  | 141 (100%) | 102 (100%) |
| **Follow-up** [Days], (Median, Interquartile Range) | 533, 489 |  |  |  | 333, 351 |  |
|  |  |  |  |  |  |  |
| **Age** |  |  |  |  |  |  |
| ≤ 50 Years | 10 (7%) |  | 33 (19%) |  | 11 (8%) | 13 (13%) |
| 51-60 Years | 45 (33%) |  | 62 (37%) |  | 37 (26%) | 30 (29%) |
| > 60 Years | 82 (60%) |  | 75 (44%) |  | 93 (66%) | 59 (58%) |
| Median Age [Years] | 62 |  | 59 |  | 63 | 62 |
| Mean Age [Years] | 62.6 |  | 59.4 |  | 64.5 | 62.6 |
| Age Range [Years] | 32-89 |  | 24-87 |  | 37-93 | 36-86 |
|  |  |  |  |  |  |  |
| **Gender** |  |  |  |  |  |  |
| Female | 26 (19%) |  | 70 (41%) |  | 26 (18%) | 31 (30%) |
| Male | 111 (81%) |  | 100 (59%) |  | 115 (82%) | 71 (70%) |
|  |  |  |  |  |  |  |
| **Smoking and Drinking Habits** |  |  |  |  |  |  |
| Non-smokers | 11 (8%) |  | 46 (27%) |  | 28 (20%) | 22 (22%) |
| Smokers (Current and Former) | 104 (76%) |  | 98 (58%) |  | 103 (73%) | 72 (71%) |
| Unknown Smoking Status | 22 (16%) |  | 26 (15%) |  | 10 (7%) | 8 (8%) |
| Range Pack/Years | 0-200 |  | 0-120 |  | 0-130 | 0-100 |
| Median Pack/Years (Smokers only) | 40 |  | 30 |  | 30 | 30 |
| Mean Pack/Years (Smokers only) | 49.2 |  | 39.8 |  | 31.2 | 31.9 |
| Unknown Alcohol Consumption | 30 (22%) |  | 38 (22%) |  | 13 (9%) | 13 (13%) |
| No Alcohol | 8 (6%) |  | 15 (9%) |  | 28 (20%) | 15 (15%) |
| Occasional Alcohol | 9 (7%) |  | 32 (19%) |  | 19 (13%) | 24 (24%) |
| Moderate Alcohol | 51 (37%) |  | 73 (43%) |  | 36 (26%) | 37 (36%) |
| Strong Alcohol | 13 (9%) |  | 6 (3.5%) |  | 5 (4%) | 6 (6%) |
| Alcoholic (Current and Former) | 26 (19%) |  | 6 (3.5%) |  | 40 (28%) | 7 (7%) |
|  |  |  |  |  |  |  |
| **Localisation** |  |  |  |  |  |  |
| Oral Cavity / Tongue / Lips | 15 (11%) |  |  |  | 38 (27%) |  |
| Oropharynx and Tonsils | 53 (39%) |  |  |  | 41 (29%) |  |
| Hypopharynx | 18 (13%) |  |  |  | 17 (12%) |  |
| Larynx | 37 (27%) |  |  |  | 33 (23%) |  |
| Others (Nasopharynx, Facial Skin) | 10 (7%) |  |  |  | 9 (6%) |  |
| CUP | 4 (3%) |  |  |  | 3 (2%) |  |
|  |  |  |  |  |  |  |
| **Tumor** |  |  |  |  |  |  |
| Primary Tumor | 107 (78%) |  |  |  | 112 (79%) |  |
| Loco-regional Recurrencel | 30 (22%) |  |  |  | 29 (21%) |  |
|  |  |  |  |  |  |  |
| **Tumor (T) Category** |  |  |  |  |  |  |
| T_is_ | 1 (1%) |  |  |  | 1 (1%) |  |
| T_1_ | 22 (16%) |  |  |  | 31 (22%) |  |
| T_2_ | 42 (31%) |  |  |  | 43 (30%) |  |
| T_3_ | 30 (22%) |  |  |  | 29 (21%) |  |
| T_4_ | 32 (23%) |  |  |  | 28 (20%) |  |
| N/A | 10 (7%) |  |  |  | 9 (6%) |  |
|  |  |  |  |  |  |  |
| **Nodal (N) Category** |  |  |  |  |  |  |
| N_0_ | 37 (27%) |  |  |  | 38 (27%) |  |
| N_1_ | 19 (14%) |  |  |  | 24 (17%) |  |
| N_2_ | 51 (37%) |  |  |  | 54 (38%) |  |
| N_3_ | 2 (1%) |  |  |  | 5 (4%) |  |
| N_x_ | 28 (20%) |  |  |  | 20 (14%) |  |
|  |  |  |  |  |  |  |
| **Distant Metastases (M) Category** |  |  |  |  |  |  |
| M_0_ | 132 (96%) |  |  |  | 138 (98%) |  |
| M_1_ | 5 (4%) |  |  |  | 3 (2%) |  |
|  |  |  |  |  |  |  |
| **Histopathological Grade** |  |  |  |  |  |  |
| G_1_ | 2 (1%) |  |  |  | 10 (7%) |  |
| G_2_ | 59 (43%) |  |  |  | 52 (37%) |  |
| G_3_ | 51 (37%) |  |  |  | 33 (23%) |  |
| N/A | 25 (18%) |  |  |  | 25 (18%) |  |
|  |  |  |  |  |  |  |
| **Lymphatic Invasion** |  |  |  |  |  |  |
| L_0_ | 64 (46%) |  |  |  | 69 (49%) |  |
| L_1_ | 28 (21%) |  |  |  | 29 (21%) |  |
| N/A | 45 (33%) |  |  |  | 43 (30%) |  |
|  |  |  |  |  |  |  |
| **Vascular Invasion** |  |  |  |  |  |  |
| V_0_ | 79 (58%) |  |  |  | 86 (61%) |  |
| V_1_ | 12 (9%) |  |  |  | 9 (6%) |  |
| N/A | 46 (34%) |  |  |  | 46 (33%) |  |

*identical patients as described by Schröck *et al.* [2]., ** including 122 patients as already described by Schröck *et al.* [2].
